# Perinatal Lamb Model of Respiratory Syncytial Virus (RSV) Infection

**DOI:** 10.3390/v4102359

**Published:** 2012-10-23

**Authors:** Rachel J. Derscheid, Mark R. Ackermann

**Affiliations:** Department of Veterinary Pathology, 2738 College of Veterinary Medicine, Iowa State University, Ames, Iowa 50011, USA; Email: rdersch@iastate.edu

**Keywords:** airways, bronchiolitis, infants, innate immunity, lambs, lung, ovine, perinatal, premature, preterm, respiratory syncytial virus (RSV)

## Abstract

Respiratory syncytial virus (RSV) is the most frequent cause of bronchiolitis in infants and children worldwide. Many animal models are used to study RSV, but most studies investigate disease in adult animals which does not address the unique physiology and immunology that makes infants more susceptible. The perinatal (preterm and term) lamb is a useful model of infant RSV disease as lambs have similar pulmonary structure including airway branching, Clara and type II cells, submucosal glands and Duox/lactoperoxidase (LPO) oxidative system, and prenatal alveologenesis. Lambs can be born preterm (90% gestation) and survive for experimentation although both preterm and term lambs are susceptible to ovine, bovine and human strains of RSV and develop clinical symptoms including fever, tachypnea, and malaise as well as mild to moderate gross and histologic lesions including bronchiolitis with epithelial injury, neutrophil infiltration and syncytial cell formation. RSV disease in preterm lambs is more severe than in term lambs; disease is progressively less in adults and age-dependent susceptibility is a feature similar to humans. Innate and adaptive immune responses by perinatal lambs closely parallel those of infants. The model is used to test therapeutic regimens, risk factors such as maternal ethanol consumption, and formalin inactivated RSV vaccines.

## 1. Introduction

Respiratory syncytial virus (RSV) is the most frequent cause of bronchiolitis in infants and children worldwide [1]. RSV was first discovered in 1955 in chimpanzees and two years later was isolated from an outbreak of respiratory disease in infants [2,3]. In the 57 years since that time, only one specific preventative measure, the humanized monoclonal antibody Palivizumab, and one therapy, the antiviral ribavirin, have been successfully developed for use in a clinical setting [4,5,6,7]. Vaccines are also not available as a result of vaccine trials of the 1960s, when enhanced disease occurred in infants, resulting in severe disease including two mortalities and hospitalization of 80% of those vaccinated [8]. Despite 50 years of research since these studies, the cellular and molecular mechanisms underlying severe disease are not fully understood [9].

Risk factors of severe RSV disease in infants include premature birth, congenital heart defects, neuromuscular deficits, Down’s syndrome, immunodeficiency/compromise, and bronchopulmonary dysplasia [9,10,11]. Of these at-risk populations, the largest group is those born premature. A survey in the United States reported premature birth rate at 12.3%, or approximately one half million [12] and worldwide, there are an estimated 13 million preterm births annually [13]. While epidemiologic evidence can identify individuals at increased risk for RSV, the mechanisms of severe disease are poorly understood. The vast majority of studies in animal models are performed in mature/adult animals, and many studies of human RSV infection are also performed in adults. Rodents, non-human primates, ferrets, cattle, and several other species have been utilized to study RSV; however, studies investigating disease in mature/adult animals fail to address the unique structure, cellularity, physiology, and immunology that contribute to the increased disease severity that can occur in perinatal infants infected with RSV. 

The perinatal (preterm and term) lamb model has been used by several investigators to study RSV disease and paramyxoviral infections of infants as lambs have similar pulmonary development and are susceptible to ruminant and human strains of RSV and parainfluenza virus-3 [14,15,16,17,18,19,20]. Lambs develop mild clinical symptoms including fever, tachypnea or increased expiratory effort (wheeze), and malaise as well as mild to moderate gross and histologic lesions when experimentally infected with bovine (bRSV) or human (hRSV) strains of RSV [14,15,16,19,20]. Bovine strains infect cattle and sheep and have much homology with hRSV strains. Disease severity is increased in preterm lambs infected with bRSV which is similar to the increased disease severity that can occur in human infants born preterm infected with human strains of RSV [19,21]. Thus, the perinatal lamb model has a strong foundation and forms a valid model for RSV as evidenced by clinical alteration of respiration as well as gross and histologic lesions.

## 2. Perinatal and Adult Lung in Regards to RSV Infection

The structural differences between infant and adult lung at the alveolar level include fewer as well as thicker-walled alveoli in the infant [22]. The primary effect of this difference is on overall decreased efficiency of gas exchange; its implication in disease is more severe clinical symptoms with less inflammation. Airway submucosal glands are present in the human trachea at 13 weeks’ gestation with rapid progressive appearance in the more distal areas; however, the gland is present but its structure is primitive, lacking the branching of more mature glands [23]. Submucosal glands are becoming increasingly recognized for their role in innate defense through expression of secretory products such as lactoperoxidase into the airways. Goblet cells form as early as 13 weeks of fetal age and contribute significantly to airway mucin production. With RSV infection of mice with the Line 19 strain, increased Goblet cell production occurs through a Toll-like receptor 7 (TLR7)-Interleukin-17 (IL-17) and IL-23 pathway [24]. The number and type of Clara cells increase in the lung during fetal and perinatal development [25,26]. As progenitor cells, Clara cells at different stages of maturity are identified by ultrastructural characteristics [27]. While Clara cell secretory protein (CSSP; also known as CC10) can be expressed as early as 10 weeks of gestation in humans [28], and almost certainly by 15 weeks gestation [25] the production of CCSP of Clara cells indicates a presence of cells but not necessarily maturity. Clara cells serve as a common progenitor for Clara and type II cells, serve a role in innate immunity through expression of immunomodulatory products such as CC10, and have a high level of cytochrome P450 enzymes that function in detoxification of xenobiotics [29,30]. Type II cells, which produce surfactant, surfactant proteins, other anti-RSV peptides and proteins, and serve as a progenitor for type I cells, are present by 20 weeks of gestation and continually form with increasing fetal age. Type II cells, Clara cells, pseudostratified ciliated cells and other airway epithelial subtypes are targets for RSV replication. Airway epithelium of the neonate, once infected by RSV, alters re-epithelialization and subsequent airway responses in the adult [31].

Cells of the innate immune system are decreased in number and often operate with decreased efficiency in infants. Dendritic cells (DCs) in neonates are reduced and the ratio of myeloid to plasmacytoid DCs is inversed as compared to adults [32]. Preterm DCs have a reduced ability to produce interferon (IFN) and decreased ability to stimulate a T helper-1 (Th1) response, although contrary to observations in mice this difference alone does not predispose infants to a Th2-skewed response [32]. Dendritic cells are integral to immune response to RSV infection and increased numbers are present in nasal washes of infants with severe RSV [33]. Neonates have a reduced proliferation pool as well as a reduced storage pool of neutrophils and those in circulation have an impaired response to chemotaxins and deficiencies at multiple stages of migration including rolling adhesion, transmigration, and lamellipodia formation. Once at the site of infection, neutrophils of infants do not function at the same level as those of adults [34]. Neutrophils of term infants have about half the amount of lactoferrin, a 30% reduction in bactericidial/permeability increasing protein (BPI), and impaired oxidase activity [34,35,36]. Neutrophils are a key component in RSV pathology, with an influx into bronchioles and, to a lesser extent, alveoli [21]. Neutrophils undergoing NETosis (a form of neutrophil apoptotic-like death mediated by pathogens) can release neutrophilic extracellular traps (NETs) composed of DNA strands, histones, and antimicrobial peptides such as cathelicidins which can entrap bacteria and have antibacterial activity [37]. NETs have not been linked with RSV, but illustrate a mechanism of innate protection induced by neutrophils and may have a role against bacterial infections secondary to RSV. Neutrophilic inflammation in any individual is always in a precarious balance between killing of pathogen and damage to native tissue. Impaired recruitment and function may be in some degree preventative of collateral damage to lung tissue, but it also decreases the efficiency of pathogen killing, while the presence of neutrophils in small airways contributes to the clinical symptoms of labored breathing (increased expiratory effort, abdominal breathing) and wheeze. 

Both the inflammatory mediators that are elicited upon RSV infection as well as the response to these can be different between neonates and adults. Co-cultures of RSV-infected DCs and T cells of either adults or umbilical cord blood elicited markedly different cytokine profiles with the primary differences attributed to differences in response to transforming growth factor beta (TGF-β) [33]. The response to chemokines in neonates varies from adult response due to the altered number and function of leukocytes as well as altered receptor expression. *In vitro* stimulation of term infant monocytes and antigen presenting cells shows decreased expression of tumor necrosis factor alpha (TNF-α), IFN-α, IFN-γ, interleukin 12 (IL-12), and IL-1β, but increased expression of IL-6, IL-8, and IL-10 [38]. 

## 3. Lambs as a Model of RSV Infection of Infants

As recently reviewed by Bem, Domachowske, and Rosenberg, a number of animal models, including chimpanzee, cotton rat, mice and cattle have been used to recapitulate aspects of the different manifestations of human RSV disease [39,40,41]. While no animal model perfectly mimics all forms of human disease, the perinatal lamb has features that are beneficial to studies with RSV (Table 1). Similar to humans, sheep are outbred allowing manifestation of the diverse nature of response to RSV that occurs in infants. Also, the ovine lung bears a close resemblance in development, airway structure and cellularity to human lung. Sheep (and cattle) have a bronchus that branches from the distal tracheal mucosa into the right cranial lobe. This bronchus can be used for fiberoptic bronchoscope inoculations if desired; however, it requires dexterity due to a near ninety degree turn as the bronchus comes off the trachea in somewhat of a perpendicular fashion. Lung lobes of lambs include a right cranial lobe (with a cranial and caudal part), a left cranial lobe, left and right middle and caudal lobes, and an accessory lobe. The lung lobes are somewhat similar in size to those of a human infant allowing substantial tissue for tissue sampling. Airway branching patterns of lambs resembles infants, unlike rodents [42] and alveolar development (alveologenesis) in human fetus and lambs begins prenatally, in contrast to the post-natal alveolar development that occurs in mice/rodents [42,43,44,45,46]. In addition, the trachea and bronchi are lined by pseudostratified ciliated epithelium and have submucosal glands which contribute to mucus secretion and lactoperoxidase production similar to human infants but is in contrast to rodents which have few or limited submucosal gland structures [42,43,44,45,46,47]. 

Airways of human and sheep express all components of an oxidative system that consists of two H_2_O_2_-generating enzymes of airway epithelia, dual oxidase (Duox) 1 and 2, along with a pseudohalide anion (thiocyanate, SCN^-^), and the enzyme lactoperoxidase (LPO) [47,48,49,50]. Hydrogen peroxide is produced by Duox enzymes onto the apical extracellular space where it reacts with SCN^-^ in a LPO-catalyzed reaction to form hypothiocyanate molecule OSCN^-^ (H_2_O_2_ + SCN^-^ → OSCN^-^) [49,50,51,52,53,54,55,56,57]. The Duox/LPO/SCN^-^ system generates sufficient OSCN^-^ to eliminate bacteria *in vitro* and *in vivo* [58,59,60]. In *in vitro *assays the addition of iodide (I^-^) to the Duox/LPO enzyme products generates hypoiodous acid (H_2_O_2_ + I^-^ → HOI) instead of the physiological product OSCN^-^ which has potent microbicidal activity against bacteria and viruses, including activity against respiratory syncytial virus (RSV), whereas OSCN^-^ exhibits little antiviral activity [61]. Inhibition of lactoperoxidase in sheep reduces clearance of bacteria such as *Mannheimia haemolytica*, and we have shown that oral administration of iodide in the form of potassium iodide greatly increases iodide levels on the surface airway liquid of lambs [62]. Thus, lambs have a fully functional Duox/LPO oxidative system [62] which can be used to assess Duox/LPO anti-RSV activity *in vivo*.

Epithelial cells of perinatal (preterm and term) lamb lung also have close similarities to human infants. This is especially important since RSV replication within bronchioles results in bronchiolitis and accumulation of neutrophils and cell debris which can occlude or partially occlude the airway lumen and also affect bronchiolar dilation and contraction. Thus, the bronchiole is both an important site for RSV replication within non-ciliated cells and type II cells at the bronchiolar/alveolar junction and also a location where RSV-induced cell injury and inflammation can greatly impair airflow and gaseous exchange. Clara cells are a major cell type of non-ciliated cells that line bronchiolar epithelium. Bronchiolar epithelium in lambs is 18–22% Clara cells which is similar to human lung, whereas mouse lung has a large (50%+) population of Clara cells in bronchiolar airway [25,26,27,45,63,64]. The Clara cells of sheep have ultrastructural features more similar to human Clara cells than most other species [27,64]. 

Damage to or dysfunction of Clara cells creates a proinflammatory environment due to the loss of their immunomodulatory secretions. Clara cells secrete large amounts of CC10 (also known as CCSP, CC16, secretoglobin, and uteroglobin) which is increased in bronchoalveolar lavage fluid (BALF) and serum during acute injury such as smoke inhalation or application of pneumotoxicants (naphthalene, 4-ipomeanol (4-IM), chloroethylene), but decreased in chronic or dysplastic airway dysfunction (asthma, chronic obstructive pulmonary disease, or bronchopulmonary dysplasia (BPD) [25,29,63,65]. CC10-deficient mice have increased inflammatory responses and persistence of RSV after infection while restoration of CC10 abrogates these effects [30] and Clara cells damaged by 4-ipomeanol enhanced RSV disease severity in calves [66]. CC10 may not only have a protective effect on Clara cells, but also stimulate development of Clara cells [63]. Clara (and type II) cells also produce substances with known anti-RSV activity including: surfactant proteins A (SP-A) and D (SP-D) which bind and opsonize RSV, beta-defensins, beta-galactoside-binding protein, and RSV receptors such as Toll-like receptor-4 (TLR-4), retinoic acid inducible gene-I (RIG-I) which triggers epithelial responses as well as inflammatory/immunomodulatory substances [29,30,67,68,69,70]. CC10 is reduced in lambs preterm which may contribute to the increased susceptibility of preterm ovine lung to RSV and the reduced levels of innate immune gene expression such as CC10 and SP-A [71]. To our knowledge, Clara cell distribution and CC10 expression has not been assessed during the ontogeny of human fetal lung. If similar to lambs, reduced Clara cell numbers may affect RSV susceptibility.

Type II cells are present in distal lung bronchioles and alveoli and proliferate upon bronchiolar and alveolar injury to replace other type II cells or alveolar type I cells [22]. Type II cells produce surfactant, surfactant proteins, defensins, cytokines, chemokines, and RIG-I and are infected by RSV in both lambs and humans [21,72]. Similar to preterm human infants, numbers of type II (CD208+) cells in preterm lamb lung are low and immature (increased glycogen retention) but are increased and have increased maturity at birth [73]. 

Preterm lambs have reduced neutrophil expression of myeloperoxidase and reduced alveolar macrophage expression of nitric oxide compared to term lambs [74]. Pulmonary dendritic cells isolated from term lamb lung differ from those isolated from adult lung in terms of antigen expression and maturation [75]. Ovine pulmonary dendritic cells from term lung support bRSV replication and have enhanced interleukin (IL)-4 and IL-10 gene transcripts [76,77]. Alveolar macrophages of term and adult sheep have differential expression of cytokines in response to bRSV and Toll-like receptor ligation [75,77]. With bRSV and hRSV A2 infection, lymphocytic infiltration of the lung includes CD4+ and CD8+ cells and tracheobronchial lymph nodes undergo marked lymphocytic hyperplasia characterized by increased paracortical and follicular lymphocytes [19,78].

In terms of innate immune and adaptive responses in developing lamb lung, ontogeny studies of fetal and perinatal lung tissue identified reduced mRNA levels of SP-A, SP-D, and sheep beta defensin-1 (SBD-1) in preterm tissues [79], while another study showed no significant difference in surfactant mRNA and very low levels of SBD-1 in prenatal and term tissue *versus* significantly higher levels in adult sheep [71]. Messenger RNA expression of Toll-like receptor-4 and -8 (TLR-4 and -8) in the lung, increases throughout gestation but for a sharp drop in TLR-4 mRNA levels in term lambs [71]. TLR-4 is associated with CD14 that recognizes the F protein of RSV [10,80]. Binding of the TLR-4/CD14 complex activates NF-κβ, eventually leading to secretion of IL-8, IL-10, IL-6, as well as increased expression of TLR-4 on epithelial cell [80]. Pulmonary TLR-7 mRNA is also significantly lower in term lambs than in preterm or adult animals [37]. TLR-7 recognizes single-stranded RNA (viral); TLR-7 mRNA is increased in infants with naturally-occurring RSV compared to infants with non-RSV bronchiolitis [71]. TLR-3 binds double stranded RNA, a replication intermediate of RSV. Term and adult lambs have similar levels of TLR-3 expression in lung, but preterm lambs have significantly higher levels [71]. Interferon gamma TNF-α, IL-6, IL-8, and monocyte chemotactic protein-1 (MCP-1) increased throughout prenatal development, peaking at birth and decreasing into adulthood [71]. Differences in TLR, cytokine and chemokine expression in perinatal lung compared to adult may affect RSV binding, replication, and immune responses.

Lambs, other ruminants, swine and other species receive maternal immunoglobulins only through ingestion of colostrum. This is because transplacental passage of immunoglobulins does not occur as in rodents and human infants. Therefore, lambs deprived of colostrum are devoid of maternal immunoglobulin and thereby lack maternal antibodies to RSV. This allows great flexibility in altering the serum levels of maternal immunoglobulins in terms lambs which lends itself well to studies assessing the role of immunoglobulins in protecting against RSV infection. In clean facilities with proper management, colostrum-deprived lambs survive and lack secondary bacterial infections unlike calves which often die in several days if not raised in gnotobiotic conditions. Thus, lambs lacking maternal immunoglobulin can be used to test vaccines without interference by maternal immunoglobulin. Because RSV is more severe in infants born preterm, and lack of antibody affinity maturation has a significant role in enhanced RSV disease [81], the extent to which fetal and preterm infants may respond to a particular vaccine (without interference by transplacental passage of maternal immunoglobulin) can be tested by vaccinated lambs *in utero*, preterm, at birth and a few weeks after birth. In addition, the effect of maternal immunoglobulin on perinatal vaccination can be further assessed by feeding lambs: colostrum lacking RSV antibody, colostrum with low neutralizing RSV antibody, colostrum with high neutralizing RSV titers, and colostrum with antibodies to formalin-inactivated RSV (FI-RSV) vaccine. Lambs can be infected with RSV by several routes, each having advantages and disadvantages. Intratracheal inoculation is a relatively rapid injection of RSV in fluid media that bypasses the nasal cavity and larynx allowing the fluid to be distributed directly into the tracheal lumen with drainage into the major bronchi. At the tracheal carinae, the inoculated fluid does not divide evenly and one side of the lung receives more or less than the other. The fluid volume inoculated can range from 1 mL to 10 mL or more. This volume of fluid delivered rushes across the tracheal luminal surface and can overwhelm the comparatively small amount of air-surface liquid and its antimicrobial contents (e.g., lactoferrin, antimicrobial peptides, oxidative products). Bronchoscopic inoculation of RSV in fluid media has the benefit of very precise delivery with a fluid volume of virus into the bronchi that is more accurately localized than intratracheal inoculation but is still is often rapidly injected resulting in a relatively large, single volume of a fluid inoculum that can locally overwhelm the air-surface liquid. Aerosolized RSV inoculum delivered using atomizer devices into the nares results in deposition of droplets in the upper and lower respiratory tract; however, these mists are often rapidly delivered and the particles (ranging from 20 to 100 um in diameter) are too large for consistent accumulation into the distal bronchioles and alveoli. Both aerosolized inoculum and nebulized mists reduce the amount of fluid volume deposited onto the nasal mucosa and thereby decrease the chance of the fluid volume of the inoculum to overwhelm the air-surface liquid volume. Nebulized mist can be delivered over a specified time and deposits RSV inoculum in the upper and lower respiratory tract allowing deposition to the bronchioles and alveoli. Thus, there is much flexibility in the type of inoculation route to deliver RSV to the lung which can be tailored to assess anti-RSV drugs, vaccines, or RSV pathogenesis. We prefer nebulization for studies that require even distribution of virus throughout the lung especially studies assessing mucosal innate immune responses.

There are some hurdles to overcome in using lambs for RSV studies. First, a vendor that can supply healthy lambs or a healthy flock is needed. Second, availability is limited to spring and fall lambing seasons; however, the fall lambing season in some areas, including the midwest area of the United States, can occur from September to December and the “spring” lambing season can last from January to June. Investigators with access to multiple vendors that differ in breeding regimens can have a very broad availability. Third, facilities for handling lambs and some expertise in ruminant anatomy, physiology and medicine is beneficial especially for studies of colostrum-deprived lambs which require a very clean environment, although most facilities for dogs or other midsized animals and a veterinarian on staff can easily allow for studies in lambs. Fourth, reagents to ovine proteins can be difficult to obtain; however, some antibodies to bovine proteins cross-react with ovine and the advent of proteome profiles can overcome these issues. Gene expression assays are not an issue with ovine samples since the ovine genome is becoming further clarified, primers and probes to bovine work well, and next-generation sequencing (NGS) allows extensive coverage. Fifth, premature lambs do not survive but preterm lambs (90% gestation) have a good survival rate [19].

**Figure 1:**
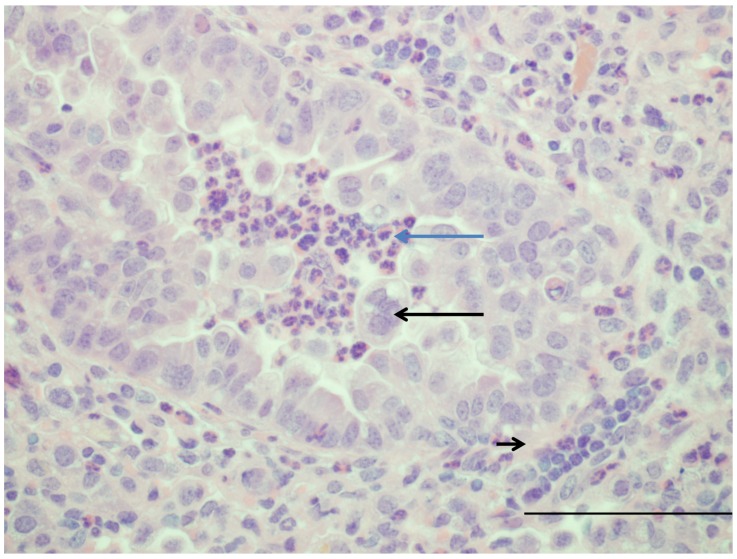
Image of lung from a lamb that at 2 days of age was infected with human respiratory syncytial virus strain, Memphis 37 by nebulizer (6.4 × 10^7^ plaque-forming units (PFU)/mL). Six days after infection, lung tissue was collected. The bronchiole has moderately thickened epithelium due to hypertrophy and proliferation of epithelial cells and within the lumen are neutrophils (blue arrow) and large syncytial cell (arrow). Around the airway in the tunica adventitia are lymphocytes (short arrow). H and E. Bar = 120 um. Manuscript in preparation.

Lambs are naturally susceptible to ovine and bovine RSV strains and can be experimentally infected with a number of strains. Respiratory syncytial virus (RSV) is in the *Paramyxoviridae* family, subfamily *Pneumovirinae*. The *Pneumovirus* genus includes human, bovine, ovine, and caprine respiratory syncytial viruses as well as murine pneumovirus (pneumonia virus of mice). The four respiratory syncytial viruses are closely related while murine pneumovirus shares less homology. Initial studies by Lehmkuhl, Cutlip, Belknap, Lapin and others determined susceptibility of lambs to bRSV experimentally and characterized histological and ultrastructural lesions as well as immunoglobulin responses to infection [14,15,16,17]. More recent studies have assessed infection by human strains of RSV (hRSV) [20,82]. There are two primary strains of RSV, A and B, with many genetically divergent substrains of each. Human RSV A2 strain replicates in lambs and causes disease but as this is a laboratory-adapted strain, other strains may have enhanced virulence [20,82]. Infection with RSV in term as well as older (6 month old) lambs will cause clinical disease exhibited as fever, listlessness, and tachypnea, as accompanied by gross and histologic lesions. Histologically, lesions caused by bRSV and strains of hRSV are similar to those seen in infants and cattle and include bronchiolitis with epithelial cell necrosis, syncytial cell formation, hyperplasia of nearby epithelium (subacutely) and infiltrates of neutrophils with occasional macrophages [15,19,20,83,84] (Figure 1). Neutrophil infiltration in lambs is associated with increased levels of IL-8 expression, which is similar to human infants; rodents do not produce IL-8 but instead express the chemokine KC which has some overlap with IL-8 in function [78]. The adventitia of infected bronchioles is infiltrated by lymphocytes (CD4+ and CD8+) and plasma cells and alveolar lumens contain small amounts of cell debris with occasional alveolar macrophages [15,19,21,78,80]. Tracheobronchial lymph nodes become enlarged due to follicular and paracortical hyperplasia [78]. Viral antigen is present in bronchiolar epithelium which contains Clara cells, occasional type II cells lining alveoli, and occasional alveolar macrophages [19,20,82,85]. Infection of term lambs with another paramyxovirus, parainfluenza-3 (PI-3), caused similar lesions and induced increased mRNA expression of sheep beta defensins-1 (SBD-1), SP-A, and SP-D that was associated with viral clearance; protein levels of SP-A did not change throughout the course of disease [34]. 

Preterm lambs can be derived by Caesarean section at 90% gestation with 80% survival for experimentation [19,74]. These lambs are not premature; however, innate immune gene responses, cellular differentiation, and responses to RSV by preterm lambs have significant differences from term lambs [71,79]. In contrast, rodents do not survive preterm birth. Infection of preterm lambs with bRSV resulted in enhanced disease with decreased viral clearance, increased viral antigen, mRNA levels RSV, and infiltration of bronchioles by neutrophils compared to term lambs [19,74]. bRSV-infected cells retrieved from lung tissue by laser capture microscopy (LCM) had increased SP-A and MCP-1 mRNA in both term and preterm lambs but expression was significantly lower in infected preterm lambs compared to term lambs [72]. These studies suggest that preterm lambs have reduced expression and responses of innate immune genes to RSV which may contribute to the increased disease severity.

The Long strain of RSV-A and type B RSV will infect lambs and cause clinically detectable RSV disease [69]. Similar to bRSV, hRSV strain A2 infected term lambs, causing fever and inducing pulmonary lesions similar to those observed with bRSV in lambs [20,78]. In these lambs at day 3 post-inoculation (p.i.) there are increased mRNA levels of TNF-α and IL-10 as well as increased TNF-α protein in lung tissue. At 6 days p.i. IFN-γ, IL-8, MCP-1, and macrophage inflammatory protein-1 alpha (MIP-1α) mRNA levels are increased in infected levels compared to controls while IFN-β, TNF-α, TGF-β, IL-10, and Regulated upon Activation, Normal T-cell Expressed, and Secreted (RANTES) mRNA are all decreased by infection with RSV. At 14 days p.i. the following mRNA levels are decreased in infected compared to control: IFN-β, IFN-γ, TNF-α, TGF-β, IL-10, IL-8, MCP-1, and RANTES [78]. Memphis 37 (M-37) is a human RSV-A strain isolated from a pediatric case and used in studies in human adult subjects [86] and can replicate and cause disease in lambs. Recent studies have shown that M37 causes similar disease to that of both bRSV and hRSV A2 strains (personal observations). Lambs inoculated with M37 grown in Vero cells had reduced disease severity compared to lambs inoculated with M37 grown in HEp2 cells; this finding is consistent with studies showing that growth of RSV in Vero cells reduces G protein expression [87]. 

**Figure 2:**
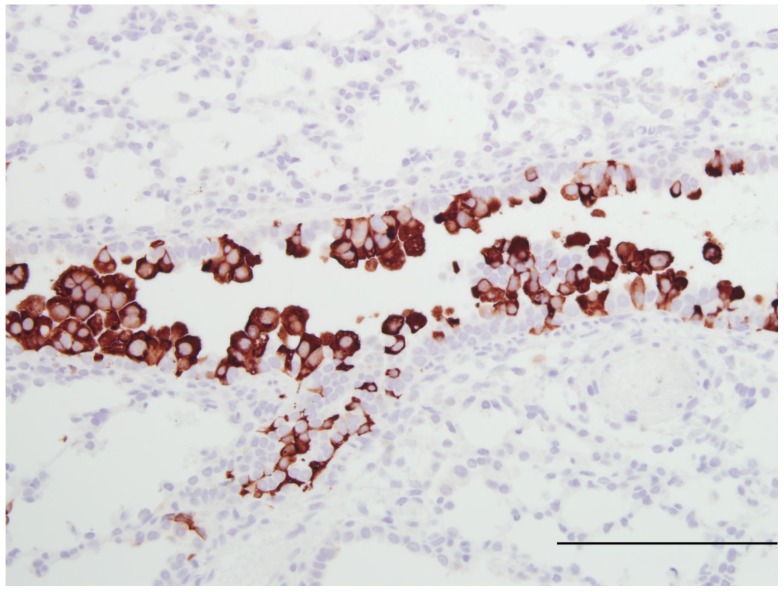
Image of lung from a lamb fed a milk replacer lacking iodide and infected at 2 days of age with human respiratory syncytial virus strain, Memphis 37 by nebulizer (6.4 × 10^7^ PFU/mL). Within the bronchiolar epithelium there is dense accumulation of RSV antigen detected by immunohistochemistry (primary antibody Meridan Biosciences). Hematoxlyn counterstain. Bar = 150 um.

**Figure 3:**
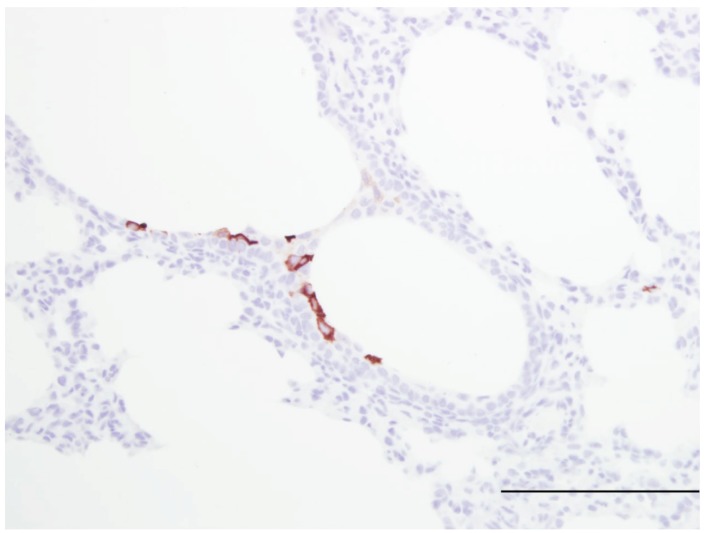
Image of lung from a lamb fed a milk replacer lacking iodide but was supplemented with iodide by gastric gavage daily (10 mg/kg body weight). At two days of age the lamb was infected with human respiratory syncytial virus strain, Memphis 37 by nebulizer (6.4 × 10^7^ PFU/mL). Within the bronchiolar epithelium there are a few cells containing viral antigen; however, significantly fewer cells are stained compared to the staining of the lung tissue from the lamb in Figure 3. Thus, with KI administration there is reduced viral antigen which is likely due to formation of iodide hypohalide (HOI) by dual oxidases and lactoperoxidase. RSV antigen was detected by immunohistochemistry (primary antibody Meridan Biosciences). Hematoxlyn counterstain. Bar = 150 um.

The lamb model has been used to test at therapeutic intervention. Vascular endothelial growth factor (VEGF) administered intratracheally before viral inoculation had a protective effect against bRSV characterized by decreased inflammation and decreased viral replication [85]. A similar protective effect of VEGF was shown using hRSV A2 and associated with increased SP-A and TLR-4 gene expression and delays in expression of anti-inflammatory mediators TGF-β and IL-10, decreasing their expression at 16 and 24 hours then increasing expression at 32 hours after VEGF administration as compared to control lambs [82,88]. The mechanism(s) by which VEGF reduces RSV severity is not known; however, VEGF increases expression of SP-A which opsonizes and aggregates RSV. VEGF also has other activities including those that may have anti-RSV effects such as: enhancement of monocyte infiltration, macrophage activation and induction of vascular leak. We are currently using the lamb model to assess efficacy of iodide enhancement of Duox/LPO-mediated innate defense (Figure 2 and Figure 3). 

**Figure 4:**
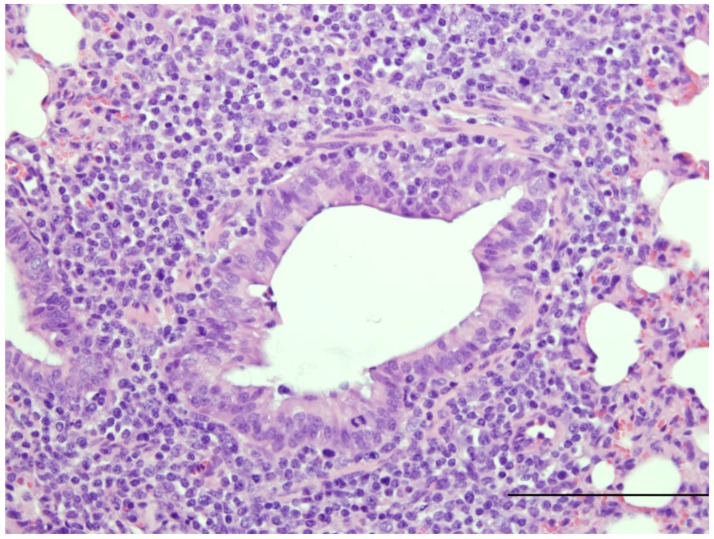
Image of lung from a lamb from a lamb that at 3 days of age was vaccinated with a formalin-inactivated respiratory syncytial virus A2 strain followed three weeks later by nebulization of human respiratory syncytial virus strain, Memphis 37 by nebulizer (6.4 × 10^7^ PFU/mL). Six weeks after nebulization, this lung was collected. The image contains a bronchiole that is mildly dilated within the tunica adventitia are dense infiltrates of lymphocytes. H and E Bar = 185 um. Manuscript in preparation.

The perinatal lamb model of RSV infection has recently been used in studies assessing the effects of formalin-inactivated RSV vaccines (FI-RSV) [89]. Our laboratory has shown that term lambs receiving one FI-RSV vaccination have increased perivascular and peribronchiolar lymphocytic infiltrates with reduced viral titers and viral antigen, as well as low serum neutralizing antibodies all of which are consistent with features that occurred in infants and other models (Figure 4; manuscript in preparation). As indicated, since the lamb can be deprived of maternal immunoglobulin, the model could be used to assess novel, promising, vaccines [89,90] without maternal immunoglobulin interference and compare such promising vaccines to those with low-affinity antibodies such as FI-RSV [81].

Maternal ethanol consumption is a risk factor for preterm birth and infants born premature have increased risk for severe RSV infection. In development of a model to assess the effects of maternal ethanol consumption at the level of a moderate drinker on the developing fetal lung, it was determined that preterm lambs born from ewes receiving ethanol during the last three weeks of pregnancy had significant reductions in SP-A and also VEGF, VEGF receptors, VEGFR1 and VEGFR2 and VEGF transcription factors hypoxia inducible factor (HIF) HIF-1α and HIF-2α but not HIF-3α [90,91]. Because VEGF is essential for lung vascular and epithelial cell growth, reductions in VEGF may contribute to the reduced SP-A expression. Since SP-A can opsonize and aggregate RSV and can also activate alveolar macrophages and infants with single nucleotide polymorphisms of SP-A have increased RSV disease severity, reductions in SP-A by exposure to ethanol *in utero* may exacerbate the susceptibility of preterm lung to severe RSV infection [51,93,94]; however, additional studies are needed to determine the extent to which ethanol exposure *in utero *may enhance RSV disease severity beyond that of preterm birth alone. Next Generation Sequencing (NGS) studies (unpublished) comparing preterm to term lambs with or without ethanol exposure *in utero* have identified transcripts with notable enhanced expression in preterm lung exposed to ethanol including genes related to (1) Inflammation, immunity and growth factors: lysozyme (19X), Secreted frizzled-related protein 2, SFRP2 which affects Wnt signaling (16X), Interleukin 12 A (9X), CXCL10 (6.8X), insulin-like growth factor-1, IGF-1 (6X), Fos (6X), IGF binding protein 5 (5.7X); early growth response protein 1, EGR1 (5X), CD28 (4X), CD4 (3.8X); (2) Metabolism/stress: leptin receptor, LEPR (5X), serotonin transporter, SERT (5X); (3) Cell proliferation inhibition: CDKN1C, p57, a Kip2 cell proliferation inhibitor (3.6X) and (4) Angiogenesis/vascularization: SERPINF1 (PEDF) was increased (4X) and this gene has anti-angiogenic properties which is consistent with findings demonstrating reductions in VEGF, VEGFR, and HIFα with ethanol exposure. Genes down-regulated by ethanol include: IL-8, TNFα, ICAM-1, all of which are up-regulated by RSV infection. Thus, ethanol alters pulmonary transcripts that may underlie susceptibility to RSV. 

**Table 1:** Features of perinatal (preterm and term) lambs integral to studies of respiratory syncytial virus (RSV) infection.

Feature	Advantage of Model	References
Prenatal alveologenesis	Similar to infants	[43,44]
Airway branching pattern	Similar to infants	[42]
Submucosal glands in airways	Similar to infants	[43,45,46,47]
Number/development of Clara cells	Similar to infants	[27,64]
Number/development of type II cells	Similar to infants	[27,64,73]
Lung size	Similar to infants	Generally known
Can survive at 90% gestation	Similar to infants	[19]
Susceptible to human RSV strains	Similar to infants	[16,20] Figure 1
Susceptible to bovine RSV strains	Permissible to RSV strains	[14,15,16,17,95]
Susceptible to ovine PI-3	Paramyxovirus susceptibility	[18]
Bronchiolitis	Similar to infants	[15,18,21,82,85]
Syncytial cell formation		[19,20] Figure 1
Neutrophil infiltration		[15,18,19,20,82,85]
CD4, CD8 T cells		[19,78]
B cells and plasma cells		[19,78]
Enhanced RSV disease severity in	Similar to infants	[19,20,74]
preterm and newborn		
Reduced immune responses preterm	Similar to infants	[71,75,76,79]
Reduced neutrophils responses preterm	Similar to infants	[74]
Dendritic cell responses to RSV	Similar to infants	[75,76,77]
IL-8 gene expression	Similar to infants	[74,78]
Functional Duox/LPO system	Similar to human/infants	[62]
Innate immune responses	Similar to infants	[34,38,71,72,74,78,79]
Adaptive immune responses	Similar to infants	[34,38,42,78]
Outbred (genetic diversity)	Similar to infants	[34,38,42,78]
Newborn lamb can be deprived of maternal	Can vaccinate newborn	Generally known
immunoglobulin (Ig)	without interference by	
	maternal Ig	
Jugular vein large and accessible	Allows placement of	Generally known
	catheter to deliver drugs	
Synchronized birth	Allows groups of lambs	Generally known
	of similar age	
VEGF reduces RSV severity	Model can test anti-RSV	[82,85]
	therapies and drugs	
Fetal lambs exposed to ethanol *in vivo *have	Model can test drugs and	[89,90]
reduced SP-A production, lung	risk factors for lung	
development, HIF 1α, HIF 2α, VEGF, and	development and RSV	
VEGFR	susceptibility	
Enhanced lymphocytic responses following	Model can study FI-RSV	Manuscript in preparation
FI-RSV vaccination	pathogenesis, mechanisms	
	and vaccines	

In summary, lambs provide a model of perinatal (preterm and term) RSV infection that is useful in exploring disease mechanisms due to their similar lung development, size, airway structure and epithelial (Clara and type II cell) composition, innate and adaptive immune response, their ability to survive if born preterm, and their ability to be deprived of maternal immunoglobulin and survive. Also, groups of lambs can be obtained for ease of experimental design. Lambs are used to study mechanisms of increased RSV disease severity in preterm lung, viral tropism to Clara and other bronchiolar epithelial cells, innate immune responses by epithelia and the Duox/LPO oxidative system, effects of anti-RSV immunoglobulins on RSV infection using colostrum-deprived lambs lacking maternal immunoglobulin, and persistent effects of RSV infection. Lambs are also valuable for use in pre-clinical trials of vaccines or therapeutics. 
